# Ceftazidime/Avibactam Monotherapy Versus Other Antibiotics: Where Do We Stand?

**DOI:** 10.3390/pathogens14111119

**Published:** 2025-11-03

**Authors:** Georgios Vougiouklakis, Constantinos Tsioutis, Nayia Vasileiadi, Konstantinos Alexakis, Nikolaos Spernovasilis, Aris P. Agouridis

**Affiliations:** 1 Department of Internal Medicine, German Medical Institute, Limassol 4108, Cyprus; georgios.vougiouklakis@goc.com.cy (G.V.); nagia.vasileiadi@goc.com.cy (N.V.); konstantinos.alexakis@goc.com.cy (K.A.); a.angouridis@euc.ac.cy (A.P.A.); 2 School of Medicine, European University Cyprus, Nicosia 2404, Cyprus; nikolaos.spernovasilis@goc.com.cy; 3 Department of Infectious Diseases, German Medical Institute, Limassol 4108, Cyprus

**Keywords:** Gram-negative, multidrug resistance, ceftazidime/avibactam, cephalosporins, carbapenems, b-lactamase, inhibitor, combination, mortality, clinical outcomes

## Abstract

The global rise of multi-drug resistant (MDR) pathogens, including the widespread resistance to beta-lactams through the production of β-lactamases, like extended spectrum β-lactamases (ESBLs), has led to the increasing use of last-line antibiotics such as carbapenems. Subsequently, the worldwide emergence of carbapenemase-producing pathogens poses a formidable challenge. The combination ceftazidime/avibactam (CAZ/AVI) has emerged as a pivotal agent in the management of multidrug-resistant Gram-negative infections. Avibactam, a novel β-lactamase inhibitor, demonstrates a wider spectrum of activity against Ambler Class A, C, and partially D β-lactamases in comparison to older inhibitors, thus enhancing the antimicrobial activity of ceftazidime against organisms producing ESBL and carbapenemases, such as oxacillinase (OXA)-type and *Klebsiella pneumoniae* Carbapenemase (KPC). This review synthesizes findings from randomized controlled trials and cohort studies, evaluating the efficacy of CAZ/AVI across diverse clinical settings, including complicated intra-abdominal infections, urinary tract infections, nosocomial pneumonia, skin and soft tissue infections, and bloodstream infections. The non-inferiority of CAZ-AVI with respect to carbapenems and superiority over polymyxins in terms of both clinical outcomes and safety are outlined, along with evidence supporting the use of CAZ/AVI in high-risk populations such as immunocompromised and critically ill patients. Overall, CAZ/AVI represents a compelling therapeutic option with favorable efficacy and safety, thus appearing as a reasonable frontline treatment for resistant Gram-negative infections.

## 1. Introduction

Over the past two decades, bacterial infections caused by multidrug-resistant (MDR) Gram-negative pathogens have emerged as a serious global public health challenge. The limited availability of effective treatment options has contributed to increased morbidity, mortality, and healthcare expenses [1]. Among the antibiotics used to combat these resistant organisms, carbapenems have long been considered drugs of last resort, along with older agents like colistin. However, the rise of carbapenem-resistant Enterobacterales (CRE) poses a critical threat. These species are common agents of both community-acquired and healthcare-associated infections. The principal mechanism of resistance in CRE is the production of carbapenemases, enzymes capable of inactivating carbapenems, thereby significantly undermining the treatment of severe infections, especially in critically ill patients [2].

Ceftazidime–avibactam (CAZ/AVI) is a novel combination of ceftazidime, a third-generation cephalosporin, and avibactam, a new, reversible β-lactamase inhibitor [3]. Unlike traditional β-lactamase inhibitors such as clavulanic acid or tazobactam, avibactam forms a covalent bond with the serine residue in the active site of β-lactamases but is not hydrolyzed in the process. Instead, it slowly dissociates, allowing the molecule to regenerate its original structure [3,4]. Avibactam exhibits inhibitory activity against Class A β-lactamases (including extended-spectrum β-lactamases [ESBLs] and *Klebsiella pneumoniae* carbapenemases [KPCs]), Class C enzymes (such as AmpC, FOX, CMY-2, and AAC-1), and Class D β-lactamases (notably OXA-48), thus in vitro restoring the antimicrobial effectiveness of ceftazidime against resistant pathogens. However, it lacks activity against metallo-β-lactamases (MBLs) like NDM, VIM, and IMP due to their lack of a serine residue at the active site, as well as against OXA-type carbapenemases found in *Acinetobacter* species [3,4].

CAZ/AVI was approved by the FDA in 2015 for use in complicated intra-abdominal infections (cIAIs) and complicated urinary tract infections (cUTIs). In 2018, approval was expanded for use in bacterial hospital-acquired pneumonia (HAP), including ventilator-associated pneumonia (VAP) [5]. Similarly, in 2016, the European Medicines Agency (EMA) approved its use for use cIAIs, cUTIs, and HAP, including VAP [6].

Early observational studies demonstrated non-inferiority between antimicrobial combinations with CAZ/AVI in comparison to meropenem combination regimens in CRE infections [7,8]. Moreover, phase 2 studies have indicated comparable outcomes regarding efficacy and safety between CAZ/AVI and meropenem in intra-abdominal infections [9]. Clinical efficacy comparable to that of imipenem–cilastatin in treating adults with complicated urinary tract infections (cUTIs) due to CRE, including ceftazidime-resistant isolates, with favorable clinical and microbiological response, has also been reported. Tolerability and safety were also displayed, supporting the potential of CAZ/AVI as a promising therapeutic option for cUTIs, including those caused by resistant pathogens [10].

Subsequently, CAZ/AVI has demonstrated favorable clinical outcomes across a range of serious infections, including complicated respiratory tract infections (cRTIs), intra-abdominal infections (IAIs), urinary tract infections (UTIs), and bloodstream infections (BSIs) caused by carbapenem-resistant (CR) *K. pneumoniae* [11]. A growing body of evidence supports the superiority of CAZ/AVI over conventional antimicrobial agents, such as carbapenems, colistin, aminoglycosides, and tigecycline, for the treatment of complicated infections. This effectiveness is evident in terms of both clinical success and patient survival, with CAZ/AVI proving effective either as part of combination therapy with conventional agents or as monotherapy. Notably, CAZ/AVI has emerged as an independent predictor of successful clinical outcomes, even after adjustment for baseline illness severity and other confounding clinical variables [11].

In phase 3 trials, CAZ/AVI has demonstrated high clinical cure rates, even in ceftazidime-resistant infections, with similar performance to best available therapy (BAT) [12]. Against non-carbapenemase-producing Enterobacterales, CAZ/AVI yielded superior microbiological response rates (80.0–85.0%) compared to BAT (57.9–64.3%) [12]. In MBL-producing isolates (e.g., NDM-1, VIM-1, VIM-2), some patients achieved clinical improvement with CAZ/AVI, although microbiological eradication often failed—reflecting avibactam’s known lack of activity against MBLs. Clinical cure rates against blaCTX-M-positive strains were also favorable (92.5–92.9%), supporting CAZ/AVI’s role in treating ESBL-associated infections [12]. Across both CAZ/AVI and comparator arms, clinical cure rates (90.6–91.1%) consistently exceeded microbiological response rates (63.0–82.6%) [12], a trend also observed in cUTIs in the RECAPTURE trials [13]. Notably, CAZ/AVI was less effective microbiologically in AmpC-producing Enterobacterales and *Pseudomonas aeruginosa*, with cure rates falling below BAT in these subgroups [12]. Notably, the non-inferiority of CAZ/AVI-based regimens in comparison to meropenem regarding efficacy and safety extends to ceftazidime-resistant pathogens, reflecting the ability of avibactam to restore ceftazidime utility [14].

Similar results have been demonstrated in critically ill, mechanically ventilated intensive care unit (ICU) patients with CRE infections [15]. In a retrospective study in Greece, patients treated with CAZ/AVI showed significantly greater improvement in SOFA score, higher rates of microbiological eradication (94.3% vs. 67.7%, *p* = 0.021), and greater clinical cure rates (80.5% vs. 52.8%, *p* = 0.010) with fewer infection relapses (2 vs. 12, *p* = 0.042) and overall survival compared to conventional agents. No significant adverse effects were observed [15]. Furthermore, emerging evidence suggests the superiority of CAZ/AVI, either in combination with other agents or as monotherapy, in solid organ transplant (SOT) recipients with CRE infections, with high survival rates and reduced mortality compared to salvage therapies [16].

In light of this evidence, CAZ/AVI has been established as a prominent agent in complicated infections due to MDR pathogens among last-resort antibiotics. Herein, we describe the comparative performance of CAZ/AVI with respect to other last-resort antibiotics based on the published literature (Table 1).

## 2. Ceftazidime/Avibactam vs. Carbapenems

One of the first studies that demonstrated the inclusion of CAZ/AVI compared to meropenem for the treatment of complicated infections was the REPROVE study, a multinational, phase 3, double-blind, non-inferiority trial involving 817 patients with cRTIs, including ventilator-associated pneumonia (VAP), mostly due to *K. pneumoniae* and *P. aeruginosa*, with 28% of isolates exhibiting non-susceptibility to ceftazidime. CAZ/AVI was shown to be non-inferior to meropenem in the treatment of hospital-acquired pneumonia (HAP) and VAP. Clinical cure was achieved in 68.8% of patients receiving CAZ/AVI compared to 73.0% in the meropenem group, with a between-group difference of −4.2% (95% CI: −10.76 to 2.46, *p* = 0.0066). Similarly, in the clinically evaluable population, cure rates were 77.4% vs. 78.1%, respectively (difference −0.7%; 95% CI: −7.86 to 6.39, *p* = 0.0007). Subgroup analyses based on renal function, prior antibiotic use, infection type, ventilator status, and APACHE II scores demonstrated no significant variation in cure rates across treatment arms. Concomitant aminoglycoside use also did not impact outcomes. Further analysis, adjusting for potentially effective concomitant antibiotic use, revealed comparable clinical cure rates. Per-pathogen clinical cure rates at the test-of-cure (TOC) visit were generally comparable between treatment groups. Interestingly, among patients infected with ceftazidime-non-susceptible pathogens in the clinically evaluable population, cure rates were 80.6% in the CAZ/VI group and 78.0% in the meropenem group, yielding a difference of 2.5% (95% CI: −16.42 to 20.74). Similar results were reproduced in patients with ceftazidime-susceptible pathogens [40].

Adverse events (AEs), mostly including diarrhea, hypokalemia, anemia, constipation, and vomiting, were reported in 75% of patients receiving CAZ/AVI and 74% in the meropenem group. Treatment-related AEs were recorded in 16% and 13% of patients, respectively. Few AEs led to discontinuation of therapy in either group. Serious adverse events (SAEs), pertaining to infections, respiratory disorders, and cardiac complications, occurred in 19% of patients in the CAZ/AVI group and 13% in the meropenem group. Notably, four patients (1%) in the CAZ/AVI group experienced SAEs (diarrhea, acute coronary syndrome, subacute hepatic failure, and abnormal liver function) deemed possibly related to the study drug [40]. These results, indicating non-inferiority with regard to the efficacy and safety of CAZ/AVI relative to meropenem, were also consistent in the Indian sub-population of the trial [37]. In this trial, CAZ/AVI demonstrated non-inferiority to meropenem, with findings consistently confirmed across multiple sensitivity and subgroup analyses, including the U.S. FDA primary endpoint and the EMA-specified criteria for HAP/VAP trials [41].

Furthermore, CAZ/AVI has been shown to attain comparable results to carbapenems regarding efficacy while maintaining a similar safety profile in cIAIs in the Asian population. Data from two early randomized, controlled, double-blind, phase 3 trials (RECLAIM-1 and RECLAIM-2), involving 1066 hospitalized adults with cIAIs due to Enterobacterales requiring invasive procedure, showed non-inferiority of CAZ/AVI plus metronidazole in comparison to meropenem. However, in patients with baseline moderate renal impairment, a trend in favor of meropenem was observed. Because dose reductions for renal impairment were greater with CAZ/AVI than with meropenem, some patients may have been underdosed early in the study, potentially influencing outcomes. Interestingly, clinical response was similar between groups with infections due to both ceftazidime-sensitive [82% for CAZ/AVI and 87.7% for meropenem, 95% CI −5.7 (−11.57 to 0.17)] and ceftazidime-resistant [83% for CAZ/AVI and 85.9% for meropenem, 95% CI −3.0 (−17.89 to 10.60)] isolates, delineating the ability of avibactam to restore ceftazidime effectiveness in real-life circumstances. Both treatment groups experienced similar AE rates, mainly gastrointestinal disorders. Mortality was slightly higher in the CAZ/AVI plus metronidazole group (2.5% vs. 1.5% with meropenem), with some deaths associated with renal impairment or cIAI progression. However, no deaths were attributed to study drugs, and no consistent trends in cause of death were observed [34]. Similar results were reproduced in the RECLAIM-3 trial [14]. Of note, in the sub-population of Indian origin with cIAIs, including those requiring ICU, CAZ/AVI plus metronidazole was proven to be a strong alternative to meropenem [36].

In the RECAPTURE trials, CAZ/AVI demonstrated clinical cure rates at the TOC visits comparable to doripenem across multiple microbiological subgroups regarding cUTIs. Overall, cure rates were 90.3% for CAZ/AVI and 90.4% for doripenem. CAZ/AVI maintained consistent efficacy in clinical cure rates (85.7–95.5%), even among patients infected with organisms harboring CTX-M, multiple ESBLs, or AmpC enzymes. Importantly, the β-lactamase genotype did not significantly impact CAZ/AVI efficacy [13]. In infections caused by *P. aeruginosa*, clinical cure was achieved in 83.3% of CAZ/AVI-treated patients and 90.0% of patients treated with doripenem. Notably, among *P. aeruginosa* strains meeting MIC-based screening thresholds, clinical cure rates dropped to 75.0% for CAZ/AVI and remained at 100.0% for doripenem. These findings suggest the overall non-inferiority of CAZ/AVI with respect to doripenem, including when it is used in the presence of resistance mechanisms [35].

In the REPRISE phase 3 trial including 333 patients from 16 countries, mostly from Eastern Europe, CAZ/AVI demonstrated non-inferiority to BAT, consisting mainly of meropenem or imipenem monotherapy (97% of cases) in treating cUTIs and cIAIs caused by CR Enterobacterales, *K. pneumoniae*, and *P. aeruginosa*. Similar overall clinical cure rates were noted between the CAZ/AVI group (90.9%; 95% CI, 85.6, 94.7) and BAT (91.2%; 95% CI, 85.9, 95.0). Microbiological eradication rates regarding cUTIs showed more favorable results in the CAZ/AVI group (81.9%; 95% CI, 75.1, 87.6) than in the carbapenem group (64.2%; 95% CI, 56.0, 71.9), possibly due to higher urine concentrations of CAZ/AVI [23]. The favorable microbiological response of cUTI patients with *P. aeruginosa* virtually resistant to CAZ/AVI (MIC > 8 mg/mL) in the CAZ/AVI group is also worth mentioning and is in agreement with clinical trials suggesting that microbiological response to an antimicrobial agent is achieved in 90% and 60% of infections caused by sensitive and resistant isolates, respectively [46]. AE profiles were comparable, reinforcing the role of CAZ/AVI as a safe option [23].

Furthermore, in a post hoc analysis of phase 3 trials regarding IAIs, it was confirmed that CAZ/AVI exhibits non-inferior clinical efficacy to meropenem, including infections caused by ESBL- or carbapenemase-producing organisms. For ESBL- or carbapenemase-producing Enterobacterales, CAZ/AVI achieved high cure rates (90.5–92.5%), slightly outperforming meropenem (84.9–85.4%). On the other hand, a lower cure rate (75.0%) compared to meropenem (86.7%) was observed in cIAIs due to AmpC-producing organisms. These findings further support the use of CAZ/AVI as a viable carbapenem-sparing agent, particularly in ESBL-dominated resistance settings [47].

In an observational study in Italy involving 102 ICU patients with KPC-*K. pneumoniae* BSIs, initiation of in vitro active antibiotic treatment within 24 h of blood culture sampling was associated with reduced 30-day mortality (29.1% vs. 63.8%, *p* < 0.001), even after risk stratification for comorbidities and regardless of the various sources of bacteremia. CAZ/AVI demonstrated non-inferiority compared to meropenem monotherapy or other regimens, including combinations of carbapenems with colistin and tigecycline. Additionally, CAZ/AVI-containing regimens were correlated with reduced frequency regarding the composite outcome of 30-day mortality or nephrotoxicity (HR 0.231 [95% CI 0.071–0.745], *p* = 0.014) compared to other regimens, especially those containing colistin [28]. Consistent results were also observed in the Chinese population in another trial [26].

In a retrospective study involving hematologic patients with CPE bacteremia, Castón et al. reported a trend to lower 30-day mortality (25% vs. 52%, *p* = 0.19) and significantly higher clinical cure rates (75% vs. 34.8%, *p* = 0.031) in patients treated with CAZ/AVI in combination with aminoglycosides or carbapenems compared to other active agents, mostly carbapenems and aminoglycosides. Despite the small sample size, this study indicates the possible superiority of CAZ/AVI in this immunocompromised population [24].

Moreover, promising prospects are reflected in studies that show comparable performance between CAZ/AVI and meropenem-vaborbactam (M-V), another powerful agent against complicated infections [17]. CAZ/AVI demonstrated non-inferiority to M-V in a multicenter, retrospective cohort study involving predominantly critically ill adults with CRE infections, mainly BSIs, RTIs, and IAIs [17]. No significant difference in successful treatment was noted between study groups (62% versus 69%, *p* = 0.49) or in 30- and 90-day mortality. However, patients who received CAZ/AVI monotherapy demonstrated a higher rate of 90-day CRE infection recurrence and resistance development, with respiratory tract infections and renal replacement therapy identified as potential risk factors. AE frequency was similar between the studied agents, despite an observed non-statistically significant trend to increased nephrotoxicity in the CAZ/AVI group, regardless of the use of additional antimicrobials [17]. On the contrary, in the TANGO II randomized trial, M-V demonstrated significantly lower rates of 28-day mortality and clinical failure compared to other regimens (monotherapy or combinations of carbapenem, aminoglycoside, polymyxin B, colistin, or tigecycline), including CAZ/AVI monotherapy, among patients with serious BSIs mostly due to CR *K. pneumoniae*. This study included patients with severe comorbidities, immune compromise, or those in ICU settings. Patients treated with M-V also experienced fewer renal AEs and had a lower likelihood of requiring additional antibiotics [43].

Favorable efficacy of CAZ/AVI seemed to extend to the pediatric population (≥3 months to <18 years) with cIAIs, mainly appendicitis complicated with perforation or abscess. The majority of the infections were polymicrobial, including anaerobes and ceftazidime-resistant Gram-negative pathogens. Efficacy and safety were similar between the CAZ/AVI plus metronidazole and meropenem groups [21]. It is notable that although avibactam is associated with phlebitis in animal studies, in this case, phlebitis seemed to be mostly associated with the increased number of infusions in the CAZ/AVI plus metronidazole group than the actual drug, in concordance with existing evidence [48].

Finally, according to a systematic review and meta-analysis, CAZ/AVI has demonstrated clinical efficacy comparable to that of carbapenems in treating various complicated infections, particularly in settings where approximately 25% of Enterobacterales produce ESBLs. Notably, in patients with cUTIs, CAZ/AVI achieved superior microbiological eradication rates. However, its safety profile warrants further investigation due to a higher observed incidence of SAEs compared to carbapenem-based therapies [49].

## 3. Ceftazidime/Avibactam vs. Colistin

Evidence from retrospective studies indicates comparable cure rates regarding CRE infections between patients receiving CAZ/AVI and those receiving colistin-based regimens. Furthermore, CAZ/AVI was associated with lower all-cause and infection-related mortality, along with a tendency toward shorter hospital stays. The results retain consistency among infections primarily involving OXA-48-producing *K. pneumoniae*, underscoring the clinical value of CAZ/AVI, particularly in regions where OXA-48 is prevalent [18,19].

Furthermore, CAZ/AVI exhibited enhanced clinical efficacy in the treatment of critically ill patients with infections caused by CREs. The CAVICOR study, a multicenter retrospective analysis conducted in Spain involving 339 patients with BSIs, RTIs, and IAIs primarily due to OXA-48 and KPC-producing Enterobacterales, demonstrated lower 30-day mortality with CAZ/AVI-based regimens (13.7% vs. 22%, *p* = 0.04). The beneficial effect on survival, particularly in patients with an INCREMENT score exceeding 7, suggests that CAZ/AVI-based regimens, including monotherapy, may represent a suitable treatment option for this high-risk population. CAZ/AVI-containing regimens were also associated with improved clinical and microbiological responses. Furthermore, the safety profile of CAZ/AVI was more favorable compared to colistin, particularly with respect to nephrotoxicity [25].

In another study, patients with primary bacteremia and IAIs exhibited the highest 30-day mortality rates (81.8% and 50%, respectively), while central venous catheter (CVC)-related BSIs were associated with significantly lower mortality (18%, *p* = 0.009). A considerable proportion of patients with primary bacteremia and IAI received colistin-based therapies (63.6% and 37.5%, respectively), and these patients experienced higher mortality rates than those treated with CAZ/AVI or alternative regimens. Although not statistically significant, this trend persisted across subgroups, including IAIs (64.3% vs. 37.5%) and CVC-related BSIs (37.5% vs. 0%) [28]. The reduced efficacy of colistin in IAIs may be attributable to its poor peritoneal penetration, delayed accumulation in peritoneal fluid, and potential inoculum effect in difficult-to-access sites. In contrast, among patients with urinary tract-associated BSIs, mortality was lower in those treated with colistin-containing regimens (33.3%) compared to those receiving other therapies (100%), possibly due to colistin’s rapid urinary excretion and high renal tract concentrations. Importantly, the composite endpoint of 30-day mortality or nephrotoxicity favored CAZ/AVI over colistin-based regimens (*p* = 0.01), aligning with previous evidence demonstrating significantly higher treatment-related nephrotoxicity with colistin [28].

Regarding CRE bacteremia, mostly with respect to OXA-48-producing isolates, CAZ/AVI exhibited superior performance over colistin regarding 14-day mortality (HR 0.22, 95% CI 0.06–0.77; *p* = 0.049). Non-inferiority was shown regarding 30-day mortality and bacterial eradication rates, although a non-statistically significant higher bacterial eradication frequency within 72 h (68,8% vs. 44.8%, *p* = 0.059) was noted. Interestingly, nephrotoxicity was similar between groups in contrast to other studies [28]; however, colistin was associated with increased ICU admission compared to CAZ/AVI (31% and 6.2%, *p* = 0.012) [30].

Interestingly, in a prospective multicenter study with serious infections, mostly BSIs, by KPC- and OXA-48-producing CR *K. pneumoniae*, CAZ/AVI proved to be significantly more beneficial than other regimens, mostly colistin, with regard to 28-day mortality (18.3% vs. 40.8%, *p* = 0.005). It is also notable that 46.3% of the CAZ/AVI group received monotherapy. Additionally, most patients were critically ill with high APACHE and ≥2 Charlson scores, requiring ICU hospitalization. Treatment with CAZ/AVI in this study proved to be not only non-inferior to colistin but also an independent factor of survival [32].

Similar results were described in another study with patients with CR *K. pneumoniae* BSI. At the time of bacteremia onset, 50% of the patients were hospitalized in the ICU. Bacteremia was either primary (26%) or secondary to IAI (46%), RTI (13%), UTI (13%), and SSTI (3%). Most isolates carried the blaKPC gene, with KPC-2 and KPC-3 subtypes identified in 76% and 24% of cases, respectively. Clinical success was significantly higher among patients treated with CAZ/AVI-based regimens compared to treatment with colistin or aminoglycosides (*p* = 0.006), including dual therapies with two in vitro active agents (*p* = 0.02). Survival rates at 30 and 90 days were higher in the CAZ/AVI group (92%) compared to other regimens (69% and 55%, respectively), with statistical significance reached at 90 days (*p* = 0.01). Notably, survival reached 100% in patients treated with CAZ/AVI plus gentamicin. Regarding nephrotoxicity, AKI rates were lowest with CAZ/AVI regimens (18%) compared to higher rates in colistin-containing regimens (57%) and aminoglycoside-containing regimens (44%). Colistin was associated with significantly more AKI than aminoglycosides. Treatment with colistin or aminoglycosides was linked to a notably higher overall AKI risk compared to regimens not containing these agents (*p* = 0.002) [38].

Favorable results have been shown in a prospective observational study, concerning mostly BSIs, and in a smaller proportion RTIs and UTIs due to CRE, primarily *K. pneumoniae*, regarding all-cause mortality after risk-stratification. Notably, significantly fewer patients in the CAZ/AVI group required additional CRE-active agents compared to the colistin group (63% vs. 94%, *p* < 0.001), reinforcing its role as a potentially more effective and safer monotherapy option for serious CRE infections [42].

In contrast, a study with a small sample of patients with multidrug-resistant Gram-negative bacteria, mainly *P. aeruginosa*, *K. pneumoniae*, and *Acinetobacter baumannii*, showed higher mortality with CAZ/AVI in comparison to colistin, which was attributed either to the presence of metal-β-carbapenemases or mutations of Ambler class A lactamases, such as CTX-M, which render CAZ/AVI ineffective [33].

## 4. Ceftazidime/Avibactam vs. Polymyxin B

Evidence suggests non-inferiority of CAZ/AVI in comparison to polymyxin B (PMB) treatment, as shown in a retrospective study involving CRE RTIs, UTIs, and BSIs. Clinical and microbiological cure rates and 30-day mortality were comparable between study groups. However, CAZ/AVI demonstrated a superior safety profile concerning nephrotoxicity (19% vs. 43%, *p* = 0.048) [29].

Another retrospective comparative analysis between CAZ/AVI and PMB-based regimens revealed superior outcomes in favor of CAZ/AVI, particularly when administered as part of combination therapy, in critically ill patients with CR *K. pneumoniae* infections [44]. CAZ/AVI combination therapy resulted in significantly higher 30-day microbiological eradication rates and lower mortality compared to monotherapy. In contrast, such a benefit was not observed in the PMB group, where monotherapy actually yielded slightly better microbiological eradication than combination therapy. When comparing both treatment cohorts, CAZ/AVI demonstrated a markedly higher microbiological eradication rate at both 14 days (51.2% vs. 26.8%; *p* = 0.001) and 30 days (80.5% vs. 32.9%; *p* < 0.001). Survival analysis also indicated a substantial reduction in 30-day all-cause mortality in the CAZ/AVI group (35.4%) compared to the PMB group (69.5%, *p* < 0.001). Regarding safety, AE was generally mild in both groups. Diarrhea was the most common AE, affecting 17.1% of CAZ/AVI patients and 12.2% of those receiving PMB (*p* = 0.377). Elevated liver enzymes (ALT/AST) were observed in 4.9% of patients treated with CAZ/AVI. Meanwhile, acute kidney injury (AKI) occurred in 8.5% of patients in the PMB group, along with a small number of Clostridium difficile infections (two cases in the PMB group vs. one in the CAZ/AVI group). Kidney function markers worsened significantly in the PMB group, whereas coagulation profiles remained stable across both cohorts [44].

Similar results were described in a real-world retrospective analysis involving 276 patients with mostly RTIs, BSIs, UTIs, IAIs, and SSTIs due to CR *K. pneumoniae* infections [45]. The CAZ/AVI group demonstrated significantly superior results in overall clinical efficacy (77.8% vs. 50.8%, *p* = 0.002), 7-day microbiological clearance (47.2% vs. 20.8%, *p* = 0.005), and overall microbiological eradication (75.4% vs. 37.8%, *p* < 0.001), while also exhibiting a notably lower incidence of acute kidney injury (AKI) compared to the PMB group (12.7% vs. 38.1%, *p* = 0.001). Interestingly, no significant differences were found in 30-day all-cause mortality between the two cohorts (19.0% for CAZ/AVI vs. 20.6% for PMB; *p* = 0.823). Regarding RTIs specifically, clinical success was higher in the CAZ/AVI group than the PMB group (67.4% vs. 58.2%), though the difference was not statistically significant (*p* = 0.196). However, microbiological clearance remained significantly higher in the CAZ/AVI group (76.3% vs. 40.0%, *p* < 0.001), and the incidence of AKI continued to be lower (13.2% vs. 38.8%, *p* < 0.001). Mortality again did not differ significantly (18.1% vs. 13.4%, *p* = 0.401). In the subgroup analysis of patients with bloodstream infections, CAZ/AVI outperformed PMB in both clinical success (76.5% vs. 46.2%) and microbial clearance rates (77.8% vs. 33.3%), both with statistical significance (*p* < 0.05). Nonetheless, mortality rates and AKI incidence did not differ between the two treatments in this subgroup [45].

Furthermore, similar results were consistent in various infections, mostly RTIs, BSIs, and SSTIs, due to CR *P. aeruginosa* in particular. However, the CAZ/AVI group experienced longer ICU and total hospital stays, potentially attributable to a higher proportion of immunocompromised patients and lower mortality rate in this group. Interestingly, the proportion of transplant recipients and immunocompromised patients was greater in the CAZ/AVI group [27].

Promising results in favor of CAZ/AVI were also documented among immunocompromised patients [20]. It is noteworthy that treatment with CAZ/AVI showed superior results compared to polymyxin-based regimens with regard to 14-day mortality (8% vs. 26% for patients receiving CAZ/AVI and polymyxins, respectively, OR 0.12, 95% CI 0.02–0.82, *p* = 0.03), cure rates, and hospitalization days in a small study involving cancer patients with not only carbapenemase-producing but also non-carbapenemase-producing CRE infections [20].

Furthermore, CAZ/AVI demonstrated superior performance in comparison to polymyxin-based regimens, in a retrospective study with 200 patients with CR *K. pneumoniae* infections, mainly cRTI, BSI, IAI, and UTI, including solid organ transplant (SOT) recipients, with regard to 30-day mortality (23.3% vs. 60%, *p* = 0.014) and 90-day mortality (35.7% vs. 86.7%, *p* = 0.003) [31]. Higher clinical cure (93.3% vs. 40%, *p* < 0.001) and microbiological eradication rates were also observed. These results were consistent between SOT and non-SOT patients, even in polymicrobial infections and ICU stay. It is worth mentioning that the majority of patients in the CAZ/AVI group had received monotherapy. These results seem promising, despite the small sample size and the fact that drug-related AE was not examined [31].

## 5. Ceftazidime/Avibactam vs. Aminoglycosides

Aminoglycosides are frequently used either as adjunctive therapy to enhance effectiveness against complicated infections or MDR pathogens or as monotherapy for pathogens with a very narrow sensitivity profile. CAZ/AVI is reported to be non-inferior and with a better safety profile than aminoglycosides. Evidence suggests that in CR *K. pneumoniae* BSI, clinical success is significantly higher among patients treated with CAZ/AVI–based regimens compared to treatment with colistin or aminoglycosides (*p* = 0.006), with superior survival rates. Regarding nephrotoxicity, AKI rates seem lowest with CAZ/AVI regimens (18%), compared to higher rates in aminoglycoside-containing regimens (44%) [38]. Comparable cure rates between CAZ/AVI and aminoglycosides have also been reported in BSIs, UTIs, RTIs, and IAIs with CRE and lower mortality rates associated with CAZ/AVI [19]. Furthermore, in a prospective multicenter study by Karaiskos et al. with serious infections, mainly BSIs, by KPC- and OXA-48-producing CR *K. pneumoniae*, CAZ/AVI demonstrated significantly superior performance regarding survival and bacterial eradication rates in comparison to aminoglycosides [32].

Notably, a combination of CAZ/AVI with aminoglycosides may accomplish optimal survival [38]. This was also observed in another study, involving critically ill patients with CR *K. pneumoniae,* where concomitant use of CAZ/AVI and amikacin proved to be beneficial in the 30-day mortality rate (*p* = 0.026) [44]. On the contrary, as analyzed above, in the REPROVE trial, one of the first studies that demonstrated the non-inferiority of CAZ/AVI with respect to meropenem in cRTIs (including VAP) concerning efficacy and safety, the synchronous use of aminoglycosides had no impact on outcomes [40].

## 6. Ceftazidime/Avibactam vs. Tigecycline

Tigecycline is another antimicrobial used as monotherapy or in combination in infections implicating MDR pathogens. Although tigecycline has been shown not to achieve adequate concentrations in circulation, rendering it a suboptimal option for BSIs, its utility in combination regimens is common in complicated infections [50,51].

Regarding BSIs due to CR *K. pneumoniae* in critically ill patients, treatment with CAZ/AVI-based regimens showed non-inferiority to tigecycline regimens. Time from blood culture collection to appropriate antibiotic therapy was independently associated with 30-day mortality, even excluding patients who received only tigecycline as active antibiotics. The sensitivity analysis showed that combination therapy with two or more in vitro active antibiotics was not associated with increased 30-day mortality (HR 1.58, 95% CI 0.69–3.62, *p* = 0.280) [28]. Similar cure rates between CAZ/AVI and tigecycline combinations have also been reported in BSIs, UTIs, RTIs, and IAIs with CRE, with lower mortality rates associated with CAZ/AVI [19].

Data from a retrospective Chinese study involving 187 patients with CRE-BSI showed 41.7% overall 30-day mortality, along with a Pitt bacteremia score, an immunocompromised status, meropenem MIC of ≥8 mg/L, an absence of source control of infection, and appropriate empirical therapy, representing independent risk factors. After adjustments for these factors, no significant differences in 30-day mortality were noted between CAZ/AVI monotherapy and combinations of CAZ/AVI with tigecycline or polymyxins. In contrast, CAZ/AVI-containing regimens exhibited significantly lower 30-day mortality compared to patients treated with tigecycline or combinations with polymyxins but without CAZ/AVI (17.1% vs. 47.4%, *p* < 0.001), with comparable differences regarding clinical failure (28.6% vs. 68.4%, *p* < 0.001). Combination therapy exhibited benefit only in treatment groups that did not include CAZ/AVI, whereas no difference in efficacy was noted between CAZ/AVI monotherapy and coadministration of other antimicrobials, indicating promising prospects for CAZ/AVI monotherapy with a favorable impact not only on efficacy but also on safety [26].

Furthermore, comparably favorable results extended to SOT patients. CAZ/AVI demonstrated significantly lower 30-day and 90-day mortality than tigecycline-based regimens, along with higher clinical cure rates in SOT patients with various infections with CR *K. pneumoniae*, which is noteworthy despite the small sample size, the retrospective study design, and the inability to analyze drug-related AE [31]. Other studies also support this evidence. As mentioned above, CAZ/AVI demonstrated significantly superior survival and bacterial eradication rates in comparison to tigecycline-based regimens in a prospective study involving critically ill patients with mainly BSIs by KPC and OXA-48 producing CR *K. pneumoniae* [32].

## 7. Conclusions

The utility of CAZ/AVI in complicated infections is newly established and holds promising prospects for clinicians. There is a growing body of evidence underscoring the excellent performance of CAZ/AVI in this setting.

It is worth considering that combination therapies are frequently utilized in complicated infections with MDR bacteria, which was also the case with most of the previously described studies. On these grounds, it was remarkable that CAZ/AVI was reported as an independent factor for better outcomes, not only as part of a composite regimen but also as monotherapy [26,42,43,44]. Furthermore, it is noteworthy that with the addition of avibactam, the activity of ceftazidime against ceftazidime-resistant isolates was restored [34]. Moreover, regarding infections due to CR pathogens, it is noteworthy that CAZ/AVI-containing regimens, including CAZ/AVI monotherapy, demonstrated non-inferiority compared to carbapenem combinations, which is also a standard approach in this setting [23,47]. Opting for CAZ/AVI-containing regimens, including CAZ/AVI monotherapy against CR pathogens, could be a useful carbapenem-sparing strategy and, in addition, could reduce the need for additional antibiotic administration and subsequently the possibility of AEs.

The emergence of CAZ/AVI resistance in the minority of studied patients raises important concerns [42]. Acquired resistance to CAZ/AVI among patients with persistent CRE infections following clinical exposure is also alarmingly reported. Resistance initially emerged in colonizing isolates from the respiratory, gastrointestinal, or urinary tracts [52]. Recurrence of infections often involved the same strains as the initial infections, indicating that CAZ/AVI may not fully eradicate CRE colonization within the gastrointestinal tract [39]. As such, the importance of performing CAZ/AVI MIC testing on CRE isolates, including colonizing strains, when considering therapy with this agent, is crucial [52]. Previous in vitro studies have linked resistance to amino acid substitutions in the Ω-loop and adjacent regions of the KPC enzyme [53]. Alarmingly, as high as 50% unsuccessful outcome rates have been associated with strains expressing KPC-3, OXA-9, and different TEM and SHV β-lactamases apart from the intrinsically avibactam-resistant class B metallo-β-lactamases. These findings underscore the need for rigorous clinical trials to assess CAZ/AVI’s effectiveness in treating CRE infections, as evidenced by clinical failures and relapses in previously unexposed and susceptible strains [7,22,54]. Moreover, newer agents such as cefepime/enmetazobactam may show promising results; however, evidence that directly compares those agents to CAZ/AVI is lacking. Clinical trials comparing CAZ/AVI to the newer agents would shed light on the treatment of resistant pathogens [55,56]. Nevertheless, the overall findings support CAZ/AVI as a valuable addition to the limited antimicrobial options available for CRE infections, demonstrating efficacy comparable to, if not exceeding, that of alternative treatment regimens, with a potentially more favorable tolerability profile [52].

The evidence regarding the overall favorable safety profile across studies is optimistic. AEs were reported in minimum frequency, the majority of which were of mild to moderate intensity, not necessitating discontinuation of treatment [54]. AE rates varied by infection type, with the lowest incidence in cUTIs and the highest in HAP/VAP. Most frequently reported AEs were not only gastrointestinal disorders like diarrhea, nausea, and vomiting but also headache and elevated liver enzymes, consistent with the known safety profile of ceftazidime [15,40,54]. The most common SAEs included pneumonia, acute kidney injury, respiratory failure, and sepsis, although only a few were considered drug-related. Hypersensitivity reactions have been documented in less than 10% of patients, which is comparable to other regimens. A notable laboratory finding was from a seroconversion Coombs test, which occurred to a higher degree with CAZ/AVI treatment against comparators. Despite this, no cases of hemolytic anemia or related clinical signs were reported [54]. Furthermore, the incidence of AKI observed with CAZ/AVI was notably lower at 10% compared to 30%, as previously reported with treatment regimens based on carbapenem, colistin, or aminoglycoside combinations [52]. Additionally, the association of CAZ/AVI with longer hospitalization duration was reported in some studies. However, the greater proportions of immunocompromised patients in patients receiving CAZ/AVI-based regimens should raise caution for misinterpretation of this observation [27,42]. Finally, no evidence of drug–drug interactions (DDIs) between CAZ/AVI and any commonly used concomitant medications has been revealed in studies [54].

Although overall clinical and microbiological success rates of CAZ/AVI are comparable to those of other antimicrobial agents, distinct advantages in treating resistant infections, particularly through superior microbiological efficacy against ceftazidime-non-susceptible pathogens, are prominent in the literature and clinical experience. Thus, the inclusion of CAZ/AVI in clinical treatment guidelines as well as in programs of antibiotic stewardship, optimizing its use and limiting the emergence of resistance, will certainly be beneficial. For that reason, CAZ/AVI offers a valuable addition to the armamentarium of clinicians against complicated infections due to resistant pathogens.

## Figures and Tables

**Table 1:** Characteristics of studies comparing the use of ceftazidime/avibactam versus other antibiotics.

First Author	Year	Country	Study	Population	Sample	Duration (Months)	Intervention		Comparator		Outcome
							Arm	Sample	Arm	Sample	
Ackley [17]	2020	USA	Retrospective Cohort	Primary bacteraemia, c-IAI, c-UTI, Pneumonia, Soft tissue	131	44	Ceftazidime/ Avibactam	105	Meropenem/Vaborbactam	26	Non-inferiority, similar clinical success (62% versus 69%, *p* = 0.49)
Alraddadi [18]	2019	Saudi Arabia	Retrospective Cohort	Primary bacteraemia, c-IAI, c-UTI, Pneumonia, Soft tissue	38	20	Ceftazidime/ Avibactam	10	Colistin, Carbapenem, Tigecycline, Aminoglycoside, Quinolone, Cotrimoxazole, Aztreonam	28	Similar clinical cure rate (80% vs. 53%, *p* = 0.14), relapse (20% vs. 3.6%, *p* = 0.1), and mortality (50% vs. 57%, *p* = 0.7)
Bandali [19]	2018	USA	Retrospective Poster	Primary bacteraemia, c-IAI, c-UTI, Pneumonia	150	108	Ceftazidime/ Avibactam	25	Aminoglycosides, Tigecycline, Colistin	125	Similar clinical cure rates (80% vs. 72%, *p* = 0.469), lower mortality (24% vs. 73%, *p* = 0.006)
Borjan [20]	2019	USA	Retrospective Poster	Primary bacteraemia	43	NA	Ceftazidime/ Avibactam	24	Polymyxin	19	Superior outcomes, lower mortality (8% vs. 26%, *p* = 0.03)
Bradley [21]	2019	Multicentre	RCT II	cIAI	83	22	Ceftazidime/ avibactam plus metronidazole	61	Meropenem	22	Non-inferiority regarding efficacy and safety in children
Bradley [22]	2019	Multicentre	RCT II	c-UTI	97	24	Ceftazidime/ Avibactam	68	Cefepime	29	Non-inferiority
Carmeli [23]	2016	Multicentre	RCT III	cUTI	333	20	Ceftazidime/ avibactam	152	Imipenem/Cilastatin or meropenem	153	Similar clinical cure rates, superiority in microbiological eradication CAZ/AVI (90.9%, 95% CI, 85.6–94.7) and BAT (91.2%. 95% CI, 85.9–95.0)
				cIAI			Ceftazidime/ avibactam plus metronidazole	12		15	Similar clinical cure and microbiological eradication rates CAZ/AVI (81.9%, 95% CI, 75.1, 87.6) vs. carbapenems (64.2%, 95% CI 56.0, 71.9)
Caston [24]	2017	Spain	Retrospective cohort	Primary bacteraemia, c-IAI, c-UTI, Pneumonia, Soft tissue	31	45	Ceftazidime/ Avibactam	8	Aminoglycosides, Carbapenems, Colistin	23	Lower mortality (25% vs. 52%, *p* = 0.19) and higher clinical cure rates (75% vs. 34.8%, *p* = 0.031)
Caston [25]	2022	Spain	Retrospective Cohort	Primary bacteraemia, c-IAI, c-UTI, Pneumonia, Soft tissue	339	66	Ceftazidime/ Avibactam	189	Best available therapy	150	Lower mortality (13.7% vs. 22%; *p* = 0.04), superior clinical response (89.4% vs. 79.3%; *p* = 0.01), and microbiological response (83.3% vs. 69.4%, *p* = 0.02)
Chen [26]	2021	China	Retrospective	Primary bacteraemia, c-IAI, c-UTI, Pneumonia, Soft tissue	187	36	Ceftazidime/ Avibactam	35	Aminoglycosides, Carbapenems, Tigecycline, Colistin	152	Lower mortality (17.1% vs. 47.4%; *p* = 0.001) and lower clinical failure rates (28.6% vs. 68.4%, *p* = 0.001)
Chen [27]	2022	China	Retrospective	Carbapenem-resistant *P. aeruginosa* infection	136	36	Ceftazidime/ Avibactam	51	Polymyxin B	85	lower 14-day (5.9 vs. 27.1%, *p* = 0.002), 30-day (13.7% vs. 47.1%, *p* < 0.001), and in-hospital mortality rates (29.4% vs. 60.0%, *p* = 0.001), along with a higher rate of bacterial eradication
Falcone [28]	2020	Italy	Retrospective	Primary bacteraemia, c-IAI, c-UTI, Pneumonia, Soft tissue	102	48	Ceftazidime/ Avibactam	13	Aminoglycosides, Carbapenems, Tigecycline, Colistin	78	Non-inferiority in mortality and bacterial eradication (*p* = 0.059)
John [29]	2019	USA	Retrospective Poster	Primary bacteraemia	117	97	Ceftazidime/ Avibactam	42	Polymyxin B	75	Non-inferiority in clinical cure rate. Reduced nephrotoxicity (19% vs. 43%, *p* = 0.048)
Hakeam [30]	2021	Saudi Arabia	Retrospective	Primary bacteraemia, c-IAI, c-UTI, Pneumonia, Soft tissue	61	40	Ceftazidime/ Avibactam	32	Colistin	29	Non-inferiority in mortality (*p* = 0.049) or bacterial eradication (*p* = 0.059), lower ICU admittance (31% and 6.2%, *p* = 0.012)
Hu [31]	2024	China	Retrospective	Carbapenem-resistant *K.* *pneumoniae* bacteraemia	200	42	Ceftazidime/ Avibactam	67	Other regimens	133	Lower mortality rate (23.3% vs. 60%, OR 0.19, 95% CI 0.05–0.69, *p* = 0.014), higher clinical cure rate (90% vs. 40%, OR 20.2, 95% CI 4.10–26.7, *p* < 0.001)
Karaiskos [32]	2021	Greece	Prospective	Primary bacteraemia, c-IAI, c-UTI, Pneumonia, Soft tissue	147	15	Ceftazidime/ Avibactam	71	Aminoglycosides, Tigecycline, Colistin	71	Superior survival rate (*p* = 0.003)
Katchanov [33]	2018	Germany	Retrospective	Bacteraemia	119	12	Ceftazidime/ Avibactam	5	Colistin, Carbapenem, Tigecycline, Aminoglycoside, Ceftolozane/ Tazobactam	114	Higher mortality
Krapp [7]	2017	USA	Retrospective	Pneumonia	44	5	Ceftazidime/ Avibactam	6	NA	38	At least 50% successful outcomes due to other non-metallo-β-lactamases
Lucasti [9]	2013	Multicentre	RCT II	cIAI	203	9	Ceftazidime/ avibactam plus metronidazole	101	Meropenem	102	Non-inferior clinical and microbiological response
Mazuski [34]	2016	Multicentre	RCT III	cIAI	1066	26	Ceftazidime/ avibactam plus metronidazole	532	Meropenem	534	Non-inferior in clinical response [91.7 vs. 92.5, −0.8 95% CI (−4.61 to 2.89)]
Mendes [35]	2018	Multicentre	RCT II	cUTI	810	22	Ceftazidime/ avibactam	393	Doripenem	417	Non-inferiority (−0.5% difference in clinical cure)
Mendes [12]	2019	Multicentre	RCT III	cUTI, cIAI	295	20	Ceftazidime/ avibactam	149	Imipenem/Cilastatin or meropenem	146	Non-inferiority (−3.5% difference)
Pillinger [8]	2017	USA	Retrospective Poster	Bacteraemia	73	69	Ceftazidime/ Avibactam	8	NA	65	Non-inferiority with other in vitro active regimens (36.8% vs. 33.3%, *p* = 0.87)
Qin [14]	2017	China, Korea, Vietnam	RCT III	cIAI	441	6	Ceftazidime/ avibactam plus metronidazole	219	Meropenem	222	Non-inferiority in clinical cure rate 93.8% and 94.0% (between-group difference, −0.2, 95% CI −5.53 to 4.97)
Rodgers [36]	2022	India	RCT III	cIAI	115	26	Ceftazidime/ avibactam plus metronidazole	56	Meropenem	59	Non-inferiority (difference 2.4%, 95% CI: −7.41 to 13.33)
Sathe [37]	2021	India	RCT III	HAP	78	33	Ceftazidime/ avibactam	36	Meropenem	42	Non-inferiority [8.9 95% CI (−12.09, 28.27)]
Shen [16]	2021	China	Retrospective	Primary bacteraemia, c-IAI, c-UTI, Pneumonia, Soft tissue	89	12	Ceftazidime/ Avibactam	9	Tigecycline, Colistin, Amikacin, Ciprofloxacin, Cotrimoxazole	61	Non-inferiority
Shields [38]	2017	USA	Retrospective Cohort	Primary bacteraemia, c-IAI, c-UTI, Pneumonia, Soft tissue	109	97	Ceftazidime/ Avibactam	13	Aminoglycosides, Carbapenems, Colistin	96	Superior clinical success (*p* = 0.006)
Sun [39]	2019	USA	Retrospective Poster	Bacteraemia, c-IAI, Pneumonia, Soft tissue	35	NA	Ceftazidime/ Avibactam	16	NA	19	Lower 30-day and 90-day mortality (*p* = 0.049 and *p* = 0.047, respectively)
Torres [40]	2018	Multicentre	RCT III	Pneumonia	817	33	Ceftazidime/Avibactam	409	Meropenem	408	Superior clinical cure rates (*p* = 0.0066)
Torres [41]	2019	Multicentre	RCT III US FDA-Specified End Points	Hospital-acquired and ventilator-associated pneumonia (HAP/VAP)	817	33	Ceftazidime/ avibactam	409	Meropenem	408	Superior clinical cure rates (*p* = 0.0066)
Tsolaki [15]	2020	Greece	Retrospective	Primary bacteraemia, c-IAI, c-UTI, Pneumonia, CNS	77	40	Ceftazidime/ Avibactam	41	Best available therapy	36	Higher rates of microbiological eradication (94.3% vs. 67.7%, *p* = 0.021), greater clinical cure rates (80.5% vs. 52.8%, *p* = 0.010)
Tumbarello [11]	2019	Italy	Retrospective	Primary bacteraemia, c-IAI, c-UTI, Pneumonia, Soft tissue	138	21	Ceftazidime/ Avibactam	104	Aminoglycosides, Carbapenems, Colistin	104	Lower mortality 36.5% vs. 55.8%, *p* = 0.005
Van Duin [42]	2018	USA	Prospective	Primary bacteraemia, c-UTI, Pneumonia, Soft tissue	137	52	Ceftazidime/ Avibactam	38	Colistin	99	Lower mortality (9% vs. 32%, *p* = 0.001)
Vazquez [10]	2012	Multicentre	RCT II	cUTI	137	19	Ceftazidime/ avibactam	69	Imipenem/ Cilastatin	68	Non-inferiority in microbiological response −1.1%, 95% CI: −27.2%, 25.0%)
Wagenlehner [13]	2016	Multicentre	RCT III	cUTI	1033	22	Ceftazidime/ avibactam	516	Doripenem	517	Non-inferiority [difference 6.7 (0.30 to 13.12)]
Wunderink [43]	2018	Multicenter	RCT	Primary bacteraemia, c-IAI, c-UTI, Pneumonia, Soft tissue	77	30	Meropenem/Vaborbactam	32	Ceftazidime/ Avibactam, Carbapenems, Aminoglycosides, Polymyxins	15	Higher mortality
Zheng [44]	2022	China	Retrospective	Carbapenem-resistant *K.* *pneumoniae* bacteraemia	164	36	Ceftazidime/ Avibactam	82	Polymyxin B	82	Higher microbiological eradication (*p* < 0.001) and better survival (*p* < 0.001)
Zhuang [45]	2024	China	Retrospective	Carbapenem-resistant *K.* *pneumoniae* bacteraemia	276	40	Ceftazidime/ Avibactam	178	Polymyxin B	98	Superior clinical efficacy (*p* = 0.002), microbiological clearance (*p* = 0.001), similar survival (*p* = 0.823)

## Data Availability

Not applicable.
